# Effect of salt stress tolerance of four pear rootstock clones on the salt resistance of their grafted and the involvement of possible mechanisms

**DOI:** 10.1038/s41598-025-31476-2

**Published:** 2026-01-14

**Authors:** Junwei Wang, Weili Zhang, Peng Han, Jianlong Liu, Yingjie yang, Jiankun Song, Wenchao Zhao, Zhigang Hou, Dingli Li, Ran Wang

**Affiliations:** 1 Lab of Pear Genetic Improvement and Germplasm Innovation, Qingdao Agricultural University, Qingdao, 266109 Shandong China; 2 Wanjingyuan Agricultural Science And Technology co.Itd, Zhucheng City, Weifang, 262200 Shandong China

**Keywords:** Pear, Clonal, Rootstock, Salt tolerance, Physiology, Agricultural genetics

## Abstract

Soil salinity affects the distribution of fruit trees and limits the development of the fruit industry. Rootstock plays an important role in improving the stress resistance of fruit trees.This study investigates the effects of four pear rootstock clones —QNA201 (*Pyrus calleryana* Decne.), QAUP-1 (*P. ussuriensis* Maxim), QingzhenD1 (*P. communis* L.× *P. bretschneideri* Rehd) and OHF40 (*P. communis* L) on the salt tolerance of the grafted variety ‘Luxiu’(*P.pyrifolia* Nakai cv.’Luxiu’*)* and explores the underlying mechanisms. Rootstocks and grafted seedlings were subjected to different concentrations of NaCl treatments (0 mM, 100 mM, and 200 mM for rootstocks; 200 mM for grafted seedlings). A number of physiological and biochemical indexes were determined, including salt injury index, chlorophyll content, photosynthetic parameters (*P*_*N*_, *E*_*T*_, *gS*), activities of antioxidant enzymes (SOD, POD, CAT), as well as contents and distribution of Na⁺ and K⁺.The results showed that QNA201 and Qingzhen D1 exhibited strong salt tolerance, with lower salt injury indexes, more stable photosynthetic performance, and more favorable distribution of Na⁺ and K⁺. The aboveground parts of rootstocks QNA201 and Qingzhen D1 accumulated less Na⁺ and could maintain a higher K⁺ level. The salt tolerance of grafted seedlings was consistent with that of the rootstocks. L/QNA201 and L/Qingzhen D1 had less salt damage, higher SPAD values, and stronger antioxidant activity. Na⁺ was concentrated in the roots and grafting bases, while K⁺ was enriched in the aboveground parts (especially in the leaves of L/Qingzhen D1). Transcriptomic analysis revealed significant differences in gene expression related to hormone regulation, ion transport, and antioxidant pathways in salt-tolerant rootstocks. QingzhenD1 displayed higher expression of genes involved in salicylic acid synthesis, auxin and ethylene-responsive transcription factors, and the antioxidant system, indicating its role in enhancing salt tolerance. These findings highlight the potential of QNA201 and QingzhenD1 as effective rootstocks to improve the salt tolerance of pear cultivars.

**Supplementary Information:**

The online version contains supplementary material available at 10.1038/s41598-025-31476-2.

## Introduction

Soil salinization is a global environmental problem, affecting over 1 billion hectares of land worldwide, with approximately 99 million hectares in China alone^1^. These saline-alkali soils alter regional microclimates by reducing vegetation cover and disrupting water cycles, further exacerbating aridification and limiting agricultural productivity^2^. For fruit crops, salt stress inhibits growth, reduces yield, and deteriorates fruit quality, posing a critical threat to the sustainability of the fruit industry^3^. However, research on rootstock-mediated salt tolerance in pear remains limited, despite its importance.

Rootstock plays an important role in improving the stress resistance of fruit trees. They regulate salt stress responses through multiple mechanisms, including restricting root uptake of toxic ions (Na⁺, Cl⁻), promoting compartmentalization of Na⁺ in roots to reduce shoot transport, maintaining K⁺ homeostasis, and activating antioxidant systems to scavenge reactive oxygen species (ROS)^4,5^. Ferreira-Silva et al.^6^, in their research on salt tolerance of cashew rootstocks, proposed that the salt tolerance induced by grafting is attributed to higher photosynthetic capacity, carbon assimilation rate, accumulation of proline, sugars and betaine, enhanced antioxidant capacity in leaves, as well as lower accumulation of Na⁺ and Cl⁻. Similarly, in watermelon, salt-tolerant rootstocks can improve the growth performance of scions under salt stress by optimizing ion distribution and enhancing photosynthetic efficiency^4^.

The initial effect of salinity stress on plant growth is osmotic stress^7,8^, which can lead to a decrease in cell membrane permeability and a decrease in plant water absorption^9^. In pear, salinity stress reduces relative water content, leaf water potential, root water absorption, transpiration rate, water retention, and water use efficiency^10^ and forces the closure of stomata during photosynthesis^11^. Cell division and cell elongation are also severely affected^12^^,^ and plant growth is inhibited in crops such as maize, rice, and silver buffalo fruit^13–15^. The adaptation of plants to osmotic stress is mainly achieved by reducing transpiration and the accumulation of osmoregulatory substances^16^. Under salinity stress, the stomatal conductance of leaves is reduced, which plays an important role in reducing transpiration water loss^17^. Betaine is a water-soluble alkaloid with strong osmotic regulation^18^. Proline is a small molecular organic compound that has been shown to have a protective effect under high salinity stress^19^.

Under salinity stress, reactive oxygen species (ROS) steadily accumulate and can destroy cell structure^20^. High concentrations of ROS can damage proteins, lipids, DNA, and carbohydrates^21^. In plant cells, ROS are mainly H_2_O_2_, O_2_^-^, and OH-, which are produced in the cytoplasm, chloroplasts, and mitochondria^22,23^. ROS change membrane permeability by inducing lipid peroxidation and protein oxidation^24^, which not only destroys membrane stability^25^ but also disrupts membrane integrity^26^. The detoxification of ROS is accomplished by the scavenging of toxic free radicals, which has a protective effect on plants^27,28^. The antioxidant defense system involves superoxidase dismutase (SOD), peroxidase (POD), catalase (CAT), monodehydroascorbate reductase (MDAR), glutathione S-transferase (GST), and ascorbate peroxidases (APX)^29,30^^15^^,^^31^^,^^32^^,^^33^^,^^34^.

Ion toxicity is also one of the important components of salinity toxicity, although most some studies suggest that osmotic stress is more serious than ion toxicity^18,35^. The adverse effects of soil salinization are related to the toxicity generated by excessive Na^+^^36–39^. Moreover, the increase in the Na^+^ content in plants often leads to the accumulation of Cl^-^ and the loss of K^+^. K^+^ is the main inorganic nutrient cation in non-halophytic plants and plays an important role in plant cell activity and stress responses^39–43^. Cl^-^ is a plant micronutrient that can regulate the osmotic pressure and turgor pressure of leaves and promote plant growth^44^. However, high concentrations of Cl^-^ have toxic effects and affect photosynthesis and plant growth^45^^,^^46^. The maintenance of cellular ion homeostasis is an important adaptive characteristic of salt-tolerant plants in response to excessive ions in the environment.

Pear is one of the most widely cultivated fruit trees globally, valued for its nutritional and economic importance^47^. Pears are mainly propagated by grafting. The pear clone rootstock can inherit the developmental stage of the mother parent, thus shortening the reproductive cycle. Clonal rootstocks have higher consistency and reproductive capacity than solid rootstocks. Through grafting management, clonal rootstocks can be used to obtain completely uniform seedlings, which can then be cultivated into solid clonal grafted seedlings that are more convenient for intensive cultivation management and bear more uniform fruit. However, little research has examined the salt-tolerance mechanism of clonal salt-tolerant rootstocks in the pear industry. The aim of this study was to examine the salt tolerance and salt tolerance mechanism of different clone pear rootstocks and their grafted seedlings. Specifically, the salt damage index, growth potential, and the ion concentration in different tissues of rootstock and grafted plants were measured in four clonal rootstocks: ‘QNA201’, ‘OHF40’ ‘QAUP-1’, and ‘QingzhenD1’.

## Materials and methods

### Material and treatment

Qinzhen D1 is a superior pear rootstock line developed and selected by the Pear Research Center of Qingdao Agricultural University, has obtained the Shandong Provincial Forest Tree Variety Certification (鲁 S-SSTPCB-018–2019).

QAUP-1 is a high-frequency regeneration elite line selected from Pyrus ussuriensis Maxim. by Qingdao Agricultural University, as documented in the published research paper^48^.

QNA201 line, derived from Pyrus calleryana selections (previously labeled generically as ‘Callery pear’ in lab studies; was officially designated in 2021 and is now undergoing PVR certification in China.

OHF40 is a rootstock variety originally selected and bred in the United States. For detailed information, please refer to the research paper^49^.

The scion variety ‘Luxiu’ is a pear cultivar bred and selected by the Pear Research Center of Qingdao Agricultural University,has been granted the National Plant Variety Rights (PVR) in China(CNA20171046.1).

The above variety names have all been approved.

The soil culture experiment was carried out at the National Agriculture and Forestry Science and Technology Incubator in Zhucheng, Shandong Province, China. The rootstocks were the self-root seedlings of cloned pear rootstocks QNA201 (*Pyrus calleryana* Decne.), QAUP-1 (*P. ussuriensis* Maxim), D1 (*P. communis* L. × *P. bretschneideri* Rehd), and OHF40 (*P. communis* L), which were grown for one year after being transplanted in vitro, selected, and bred by Qingdao Agricultural University. The transplanting substrate consisted of nutrient soil, vermiculite, and perlite in a mixing ratio of 3:1:1. For the hydroponic experiment, self-rooted seedlings of QAUP-1 and Qingzhen D1, which were three months old after transplanting from test-tube plantlets, were selected as experimental materials for the transcriptome experiment.The hydroponic experiment was conducted in the hydroponic room of Qingdao Agricultural University, with a temperature of 25℃, humidity of 65%, and light intensity of 3000 lux.

Standardized grafting techniques and clear success criteria ensure functional physiological connections between rootstock and scion for coordinated salt stress responses, while minimizing variability from graft incompatibility to accurately assess their contributions to salt tolerance. In May 2020, the scions of cv. Luxiu were grafted on QNA201, QAUP-1, Qingzhen D1, and OHF40 using the cut-grafting method. The criteria for successful grafting were that the graft union was completely healed without necrosis, the scion had sprouted new shoots with at least 3 functional leaves unfolding normally, and this state remained stable for more than 14 days. In the result, Luxiu uses ‘L’ instead.

For soil culture experiment, rootstock seedlings and grafted seedlings of uniform growth were randomly selected for the experiment, with 0 mM NaCl solution serving as the control. rootstocks were treated with 100 mM and 200 mM NaCl solutions, while grafted seedlings were treated with 200 mM NaCl solution. The 100 mM NaCl treatment was applied directly at the target concentration on Day 1. The 200 mM NaCl treatment a gradual increase in salt concentration: Each pot was irrigated with 1000 mL of 100 mM NaCl solution on Day 1, followed by an additional 1000 mL of 100 mM NaCl solution on Day 3 to reach the final concentration of 200 mM.. Stock and grafted seedlings were treated continuously for 21 d. Management practices were consistent throughout the trials, and regular watering was performed to balance evaporation. For the hydroponic experiment, Pear rootstock seedlings with good and uniform growth were selected, and their roots were repeatedly rinsed with clean water until clean. Then, they were transplanted into foam boxes containing half-strength Hoagland nutrient solution (the seedlings were irrigated with Hoagland nutrient solution of the same concentration during their growth period). After 10 days of cultivation, the experiment was carried out in half-strength Hoagland nutrient solution with 0 mM NaCl as the control and 200 mM NaCl as the treatment.

### Determination of the injury index

After 21 d of salt treatment, a scoring system was used to evaluate the salinity sensitivity: 0, normal green without salinity injury symptoms: 1, mild salinity damage with some leaf tips, margins, or veins turning yellow; 2, moderate salinity damage, with about half of leaf tips and margins scorched; 3, severe salt damage, with most leaf tips and leaf margins scorched, damaged, or deciduous; and 4, very severe salt damage, branch dead, leaf fall, and even death^50^. An index was then calculated as:Salt injury index (SI) = (Σ scores)/(maximum score × n) × 100 % (where “Σ scores” is the sum of scores over n plants per treatment combination, and the maximum score is 4)

### Determination of the chlorophyll content

Six plants were selected from each treatment, and the 5th to 7th leaves of selected plants were assayed by a chlorophyll analyzer SPAD-502 CKONICA U/MINOLTA (Japan). Three readings were taken for each leaf sample.

### Determination of photosynthetic parameters

The 5th to 7th leaves of three plants were selected from each treatment and the photosynthetic parameters were measured by a portable photosynthetic/fluorescence assay system (Hansateck CIRAS-3, Norfolk, England) from 9:00 to 10:00 am. The light intensity was 1200 μmol m^−2^ s^−1^, and the concentration of CO_2_ was 385 μmol mol^−1^. Measurements included net photosynthetic rate (*P*_*N*_), transpiration rate (*E*_*T*_), and stomatal conductance (*gS*).

### Activities of SOD, POD, and CAT

The mature leaves of the 5th to 7th leaves of the plants were collected between 8:30 a.m and 10:30 a.m. The extraction solution was 100 mM phosphoric acid buffer containing 1 mM EDTA, 0.1% Triton-X-100, and 1% PVP adjusted to pH 7.0. SOD, POD, and CAT activities were measured following Frederick and Landesberg^51^. The known SOD activity unit was expressed as an enzyme activity unit that inhibited the photoreduction of nitrogen-blue tetrazole by 50%. It was expressed as 0.01 POD activity unit (U) of A470 change per min and as 0.1 CAT activity unit (U) of A240 change per min.

### Determination of Na^+^ and K^+^ content

After 21 d of NaCl treatment, three plants were sampled from each treatment, and their roots, stems, and leaves were dried to constant weight. A dry sample of 0.5 g was prepared and added with 10 mL of concentrated nitric acid and 2 mL of perchloric acid for digestion at 180℃, and then the sample was dissolved to 50 mL at constant volume. An atomic absorption spectrophotometer (AAanalyst 100, PE, USA) was used to determine the Na^+^ and K^+^ concentrations.

### Statistical analysis

SPSS12.0 software was used for statistical analysis. One-way and two-way analysis of variance ANOVA was used to assess the significance of differences. Tukey’s honestly significant difference (HSD) test was used for post-hoc multiple comparisons to determine specific differences between groups, with a significance level set at *P* < 0.05.

## Results

### Tolerance of four rootstocks to salinity stress

#### Changes of salinity injury index

**Fig. 1:**
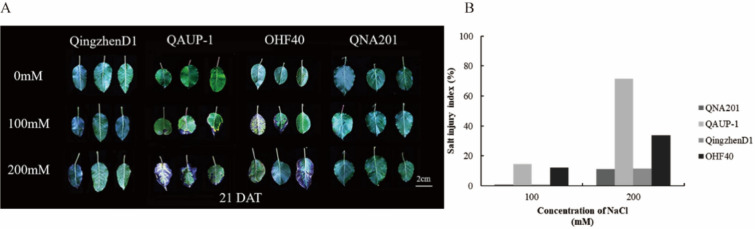
Symptom of salinity injury in different rootstocks leaves after 21 days of NaCl treatments,Salt Damage Phenotypes in Leaves of Different Rootstocks (**A**), Salt Damage Index Statistics (**B**). DAT: Day after treatments.

As shown in Figure 1 (A and B), when 4 kinds of rootstock plants were treated with 100 mM NaCl solution, QAUP-1 and OHF40 firstly exhibited salinity injury in leaves and high injury index, and QNA201 and QingzhenD1 showed no obvious toxic signs. When rootstock plants were treated with 200mM NaCl, QAUP-1 and OHF40 exhibited more serious toxic symptoms and QNA201 and QingzhenD1 just showed slightly injury symptom. The order of salt tolerance of four rootstocks was as the following: QNA201 and QingzhenD1>OHF40>QAUP-1.

#### Changes of photosynthesis

##### Changes in SPAD

**Fig. 2:**
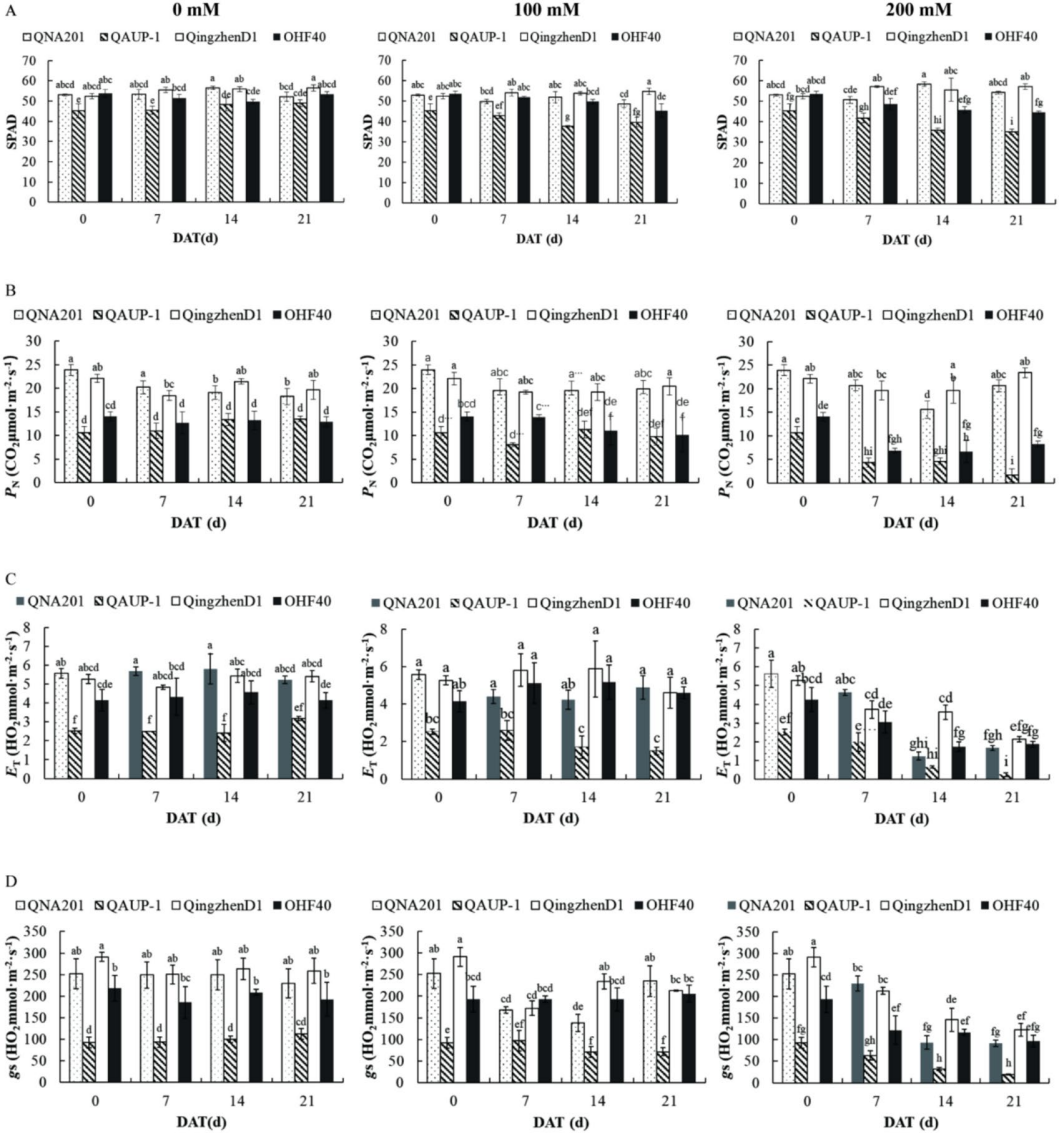
Comparison of the physiological indicators of different rootstocks treated with NaCl at 21 d. From left to right was 0 mM, 100 mM, and 200 mM. Chlorophyll content (**A**), Net photosynthetic rate (**B**), Stomatal conductance (**C**), Transpiration rate (**D**). DAT: day. after treatment. n=3 for all groups. The bars represented the SE. Bars with different lowercase letters indicated statistically significant differences at *P<0.05* based on two-way ANOVA.

The SPAD value is an indicator reflecting the chlorophyll content in leaves. Under 0mM salt stress, it can be observed that QingzhenD1 and QNA201 had relatively high initial SPAD values, while QAUP-1 had the lowest initial value. Under 100mM salt stress, the main effects of time (F=71.921, *P* < 0.01), variety (F=453.769, *P < 0.01*), and their interaction (F=28.277, *P* < 0.01) were all extremely significant. The SPAD value of QAUP-1 decreased most significantly at 7 days; although it recovered slightly in the later period, it still remained the lowest among all clones. In contrast, the SPAD values of QingzhenD1 and QNA201 remained relatively stable, showing no significant downward trend compared with the control group. Under 200mM salt stress, the SPAD values of all clones were affected. The results showed that the main effects of time (F=30.613, *P* < 0.01), variety (F=847.886, *P* < 0.01), and their interaction (F=63.172, *P* < 0.01) were all extremely significant. QAUP-1 suffered the most severe chlorophyll degradation, while QingzhenD1 and QNA201 performed well, and OHF40 showed a moderate decline (Figure 2A, Table S1).

##### Changes in *P*_*N*_

Under 0mM salt stress, the main effects of treatment time (F=9.678, *P* < 0.01), rootstock variety (F=241.350, *P* < 0.01), and their interaction (F=8.454, *P* < 0.01) were all extremely significant. Among the clones, QNA201 and QingzhenD1 had relatively high *P*_*N*_ values, while QAUP-1 and OHF40 had lower ones. Under 100mM salt stress, the main effect of treatment time was significant (F=20.160, *P* < 0.05), and the main effect of rootstock variety (F=352.184, *P* < 0.01) as well as the interaction between time and variety (F=28.277, *P* < 0.01) were extremely significant. Among them, the *P*_*N*_ value of QAUP-1 decreased most obviously, OHF40 showed a moderate decline, and the *P*_*N*_ values of QNA201 and QingzhenD1 remained relatively stable. Under 200mM salt stress, the main effects of time (F=73.824, *P* < 0.01), variety (F=796.127, *P* < 0.01), and their interaction (F=12.018, *P* < 0.01) were still extremely significant. QAUP-1 suffered the most severe degradation of photosynthetic capacity, with its *P*_*N*_ value only 15.4% of the initial value, while QingzhenD1 and QNA201 performed well (Figure 2B, Table S1).

##### Changes in *E*_*T*_

Under 0mM salt stress, the main effect of time was not significant (F=0.086, *P* > 0.05), indicating stable *E*_*T*_ values under non-stress conditions. The main effect of variety was extremely significant (F=20.748, *P* < 0.01). QingzhenD1 and QNA201 maintained relatively high *E*_*T*_ values, OHF40 showed an intermediate level, and QAUP-1 had the lowest *E*_*T*_ value. Under 100mM salt stress, the main effect of time was still not significant (F=0.794, *P* > 0.05), but the interaction effect between time and variety was significant (F=1.182, *P* < 0.05). QAUP-1 exhibited a continuous downward trend, while the *E*_*T*_ values of QingzhenD1 and QNA201 remained relatively stable, with no significant differences compared with the control group. Under 200mM salt stress, the main effects of time (F=22.958, *P* < 0.01), variety (F=12.885, *P* < 0.01), and their interaction (F=1.426, *P* < 0.01) were all extremely significant. QAUP-1 had the most significant decline, with its *E*_*T*_ value only 9.9% of the initial value; OHF40 showed a moderate decline, and QNA201 and QingzhenD1 had relatively mild declines (Figure 2C, Table S1).

##### Changes in *gS*

Under 0mM salt stress, the main effect of time was not significant (F=492.201, *P* > 0.05), while the main effect of variety was extremely significant (F=66016.799, *P* < 0.01). QingzhenD1 had the highest initial *gS* value, followed by QNA201 and OHF40, and QAUP-1 had the lowest. Under 100mM salt stress, the main effects of time (F=8026.524, *P* < 0.01), variety (F=55373.344, *P* < 0.01), and their interaction (F=3297.088, *P* < 0.01) were all extremely significant. QNA201 exhibited a recovery trend after 14 days of treatment; QingzhenD1 also maintained a relatively high *gS* value, while QAUP-1 remained at a continuously low level. Under 200mM salt concentration, the main effects of time (F=40595.139, *P* < 0.01), variety (F=45414.556, *P* < 0.01), and their interaction (F=2571.003, *P* < 0.01) were all extremely significant. QAUP-1 had the most severe decline, while QingzhenD1 and QNA201 performed well (Figure 2D, Table S1).

#### Changes of the Na^+^ and K^+^ content in different rootstocks

**Fig. 3:**
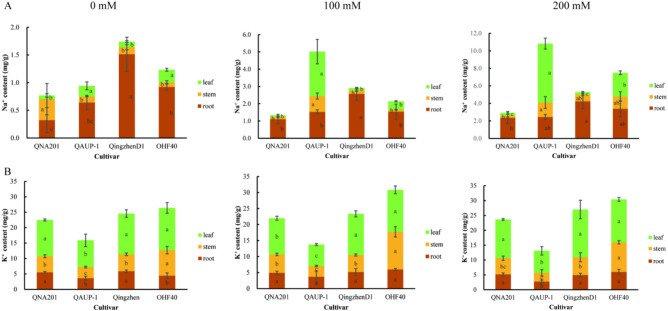
Determination of the Na^+^ and K^+^ content in the roots, stems, and leaves of different rootstocks after 21 d of NaCl treatment. From left to right: 0 mM, 100 mM, and 200 mM. n=3 for all groups. Na^+^ content in the roots, stems, and leaves (**A**), K^+^ content in the roots, stems, and leaves (**B**).The bars represented the SE. Bars with different lowercase letters indicated statistically significant differences at *P<0.05* based on one-way ANOVA.

The total Na^+^ content in rootstocks increased with the increase of NaCl treated concentration, but the accumulation of Na^+^ in roots, stems and leaves was unbalanced in different rootstocks. As shown in Figure3, under the 100 mM NaCl treatment, the total Na^+^ content of QAUP-1 raised 5.33.times as against the control, and the other three rootstocks raised similarly, between 1.67 - 1.74 times. The proportion of Na^+^ content in the roots of 4 rootstocks, QingzhenD1 was the highest(88%), followed by QNA201(82%) and OHF40(72%), and QAUP-1(30%)the lowest. The total K^+^ content in QAUP-1 was significantly lower than that in the other 3 rootstocks, and it was higher in leaves than that in roots and stems (*P* < 0.05). When the concentration of NaCl treated increased to 200 mM, the total Na^+^ content of QAUP-1 leaves increased to 11.49 times as compared with the control, followed by OHF40 (6.08 times), QNA201 (3.79 times) and Qinganzhen D1 (3.04 times). The pecentage of Na^+^ content in the roots of QNA201 and Qingzhen D1 roots still maintained a high concentration of 81% and 80%, respectively, followed by OHF40 (45%) and QAUP-1 was the lowest (23%). The results indicated that QAUP-1 had the strongest ability to absorb and transport Na^+^ to the aboveground parts, followed by OHF40. QNA201 and QingzhenD1 had the least Na^+^ absorption and a low transport ratio to the ground after absorption(Figure 3A and Table S1).The experimental results indicate that as the NaCl concentration increases, the K^+^ content in both QNA201 and Qingzhen D1 also rises. This suggests that the increase in K^+^ can partially alleviate the effects of salt stress, providing the plants with enhanced adaptability(Figure 3B and Table S1).

### Effects of 4 rootstocks on the salt tolerance of grafted variety’Luxiu’

#### Effects on the salinity injury index

**Fig. 4:**
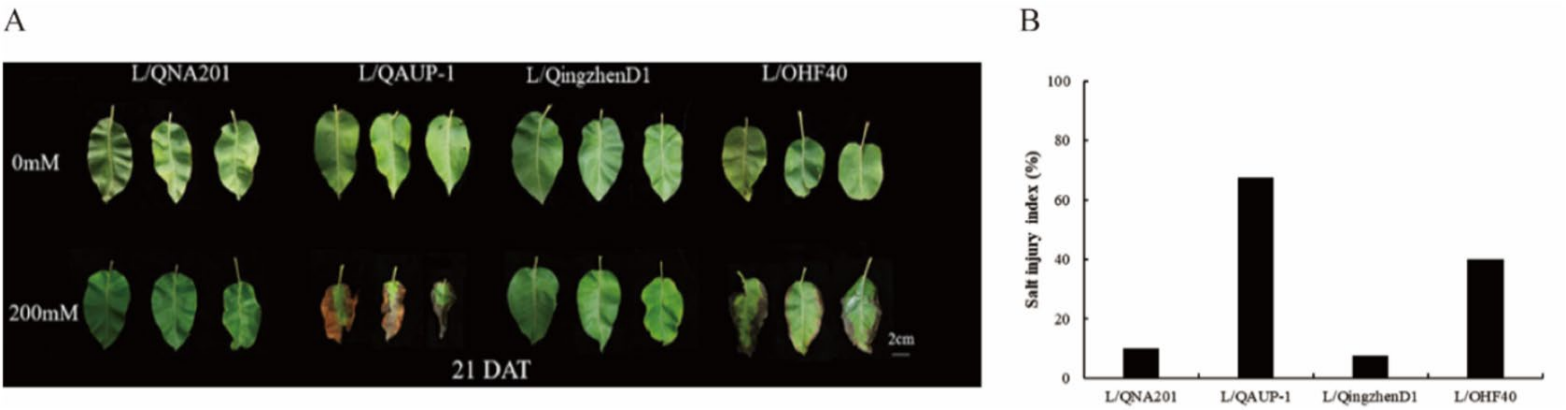
Symptom of salinity injury in leaves of ‘Luxiu’ grafted on the four rootstocks after 21 days of NaCl treatments. DAT: Day after treatments.

The variety ‘Luxiu’ were grafted on the four rootstocks and the effect of different rootstocks on the salinity injury index was shown in Figure 4 (A and B), when the grafted plants treate with 200 mM NaCl. The leaves of ‘Luxiu’ grafted on QAUP-1(L/QAUP-1) exhibited the most serious injury which was indicated by the degree of leaf scorch, followed by L/OHF40. L/Qingzhen D1 and L/QNA201 exhibited the least salt injury and no obvious signs of injury. The results were consistent with the salt tolerance of rootstocks analyzed earlier.

#### Effects on the SPAD valus

**Fig. 5:**
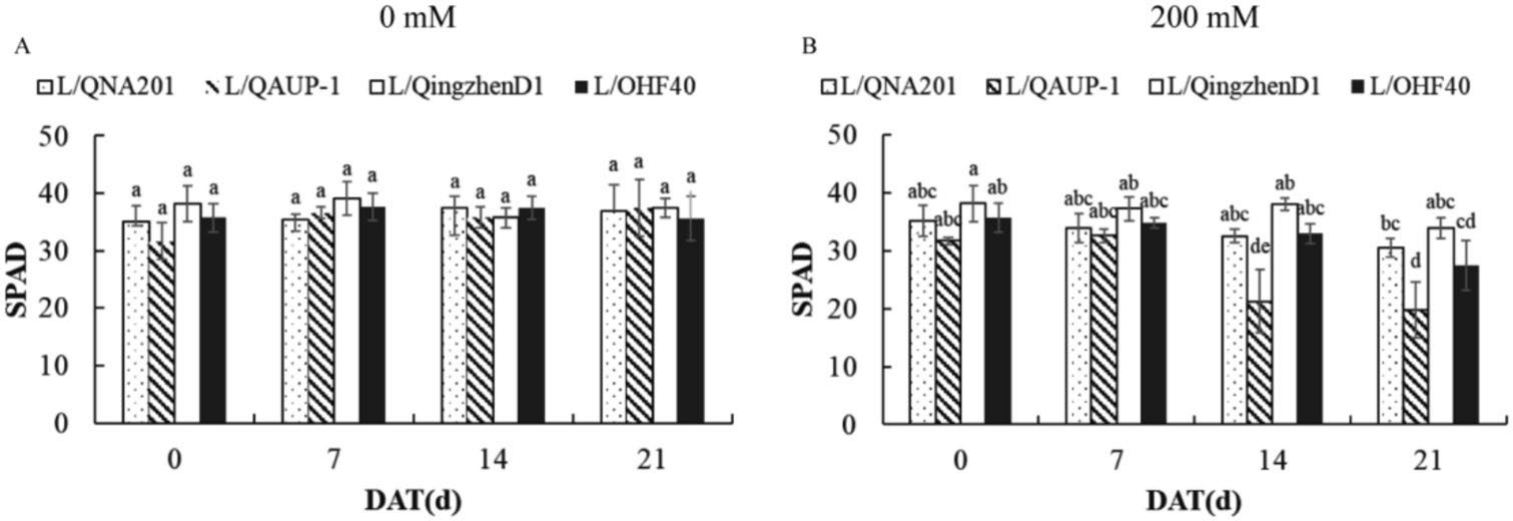
Changes of SPAD value of ‘Luxiu’ grafted on the four rootstocks during treatment of 200mM NaCl. Salt Damage Phenotypes in Leaves of Different Rootstocks (**A**), Salt Damage Index Statistics (**B**).n=3 for all groups. The bars represent the SE. Bars with different lowercase letters indicate statistically significant differences at *P<0.05* based on two-way ANOVA.

Salinity stress also affected the chlorophyll content of grafted seedlings.Under 0 mM salt treatment, the main effects of treatment time (F=1.890, *P* > 0.05, grafting combination (F=7.848, *P* > 0.05), and their interaction effect (F=6.575, *P* > 0.05) all did not reach a significant level (Table S2). This indicated that under non-stress conditions, neither the treatment duration nor the rootstock type had a significant impact on the chlorophyll content of ‘Luxiu’ grafted seedlings.Under 200 mM salt treatment, the main effects of treatment time (F=140.280, *P* < 0.01) and grafting combination (F=234.410, *P* < 0.01), as well as the interaction between the two factors (F=20.429, p<0.05), were all significant (Table S2). Among the grafting combinations, L/QingzhenD1 exhibited the least chlorophyll loss. Although the total decline of L/QNA201 was lower than that of L/QingzhenD1, it still maintained a relatively high chlorophyll level. L/OHF40 showed a moderate decline, with a 23.1% decrease compared to the initial value. L/QAUP-1 was the most sensitive to salt stress, with the greatest decrease in SPAD value (Figure 5A and 5B).

#### Effects on antioxidant enzymes

**Fig. 6:**
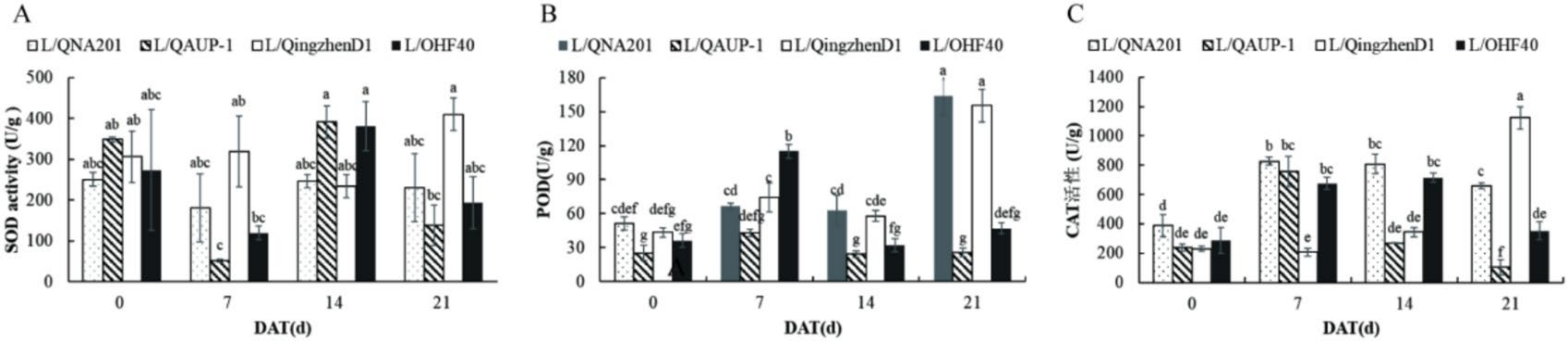
Activity of antioxidant enzymes of different grafted seedlings treated with salt at 21 d. SOD (**A**), POD (**B**), and CAT (**C**). n=3 for all groups. The bars represent the SE. Bars with different lowercase letters indicate statistically significant differences at *P<0.05* based on two-way ANOVA.

The antioxidant enzymes represented by SOD, POD and CAT of the leaves in different stions were analysed. L/QNA201 and L/Qingzhen D1 showed the highest antioxidant capacity. L/QAUP-1 was the most vulnerable to salinity stress (Figure 6 and Table S2).

For SOD activity, the results showed that the main effect of salt treatment time was extremely significant (F=50919.330, *P* < 0.01), the main effect of grafting combination was significant (F=21309.094, *P* < 0.01), and the interaction effect between the two factors was extremely significant (F=28609.752, *P* < 0.01).SOD activity of L/QNA201 recovered to its original activity after decreasing at 7 d, and L/Qingzhen D1 changed slowly in the early stage and increased significantly at 21 d. The pattern of the SOD activity in L/OHF40 and L/QAUP-1 was the same: both decreased at 7 and 21 d (Figure 6A, Table S2).

For POD activity, the results indicated that the main effects of salt treatment time (F=9128.766, *P* < 0.01) and grafting combination (F=8217.907, *P* < 0.01), as well as their interaction effect (F=3778.821, *P* < 0.01), were all extremely significant. POD activity in L/QNA201 slightly increased at 7 d, remained stable at 14 d, and increased sharply at 21 d. POD activity in L/Qingzhen D1 increased at 7 d and slightly decreased at 14 d. Both L/QNA201 and L/OHF40 had the highest POD activity at 7 d and then decreased. The POD activity in L/OHF40 was higher than that in L/OHF40 after stress (Figure 6B, Table S2).

For CAT activity, the results showed that the main effects of salt treatment time (F=258359.655, *P* < 0.01) and grafting combination (F=209257.106, *P* < 0.01), along with their interaction effect (F=272443.188, *P* < 0.01), were all extremely significant. The CAT activity in L/QNA201 and L/OHF40 increased at 7 d, remained stable at 14 d, and continued to increase at 21 d. The CAT activity in L/OHF40 was always lower than that in L/QNA201, but its decrease was greater than that in L/QNA201. L/Qingzhn D1 showed little change in CAT activity in the early stage but increased sharply at 21 d (*P* < 0.05), and L/QAUP-1 CAT activity increased sharply at 7 d and then decreased rapidly (Figure 6C, Table S2).

#### Effect on content and distribution of Na^+^and K^+^

**Fig. 7:**
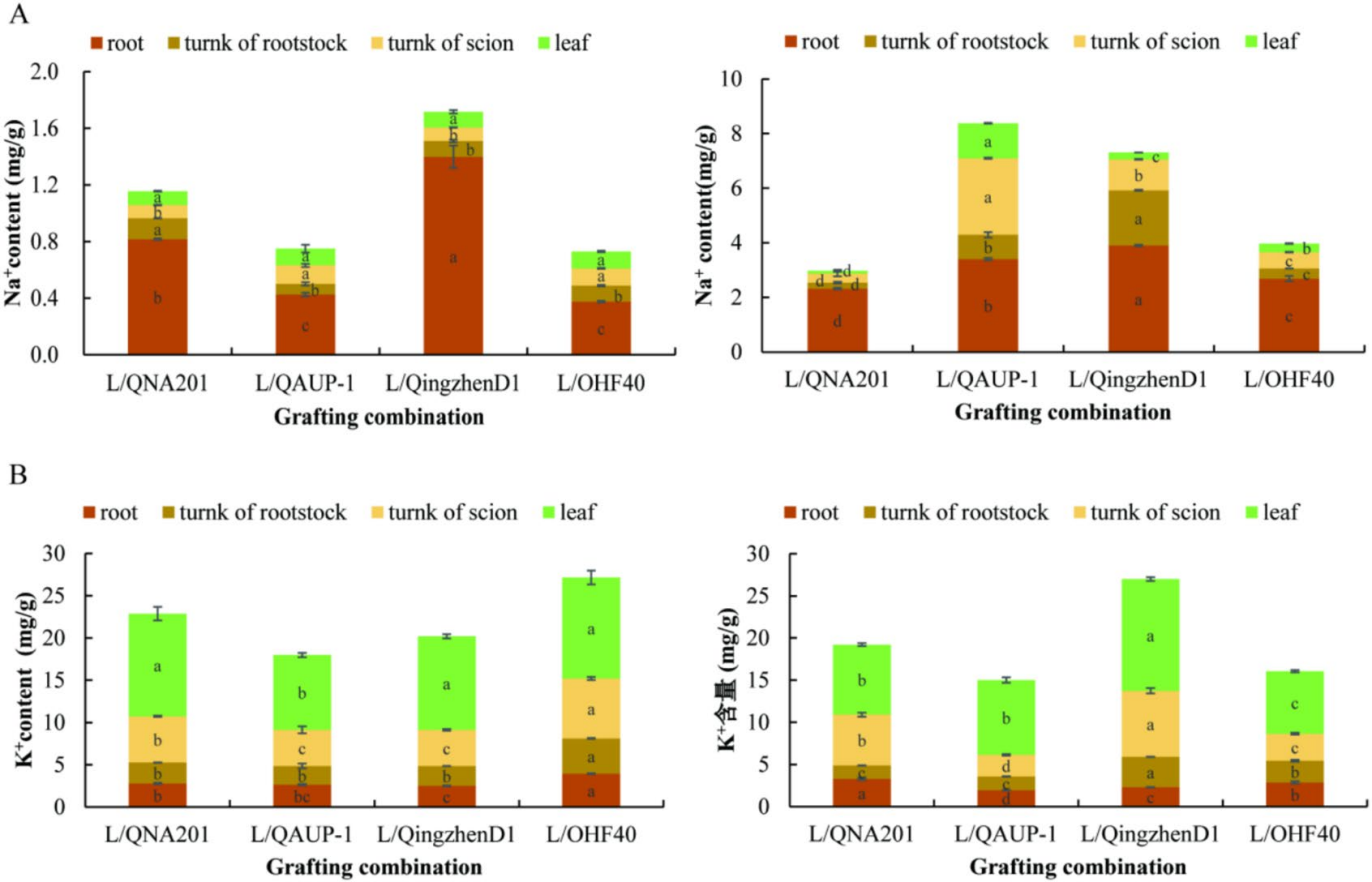
content and distribution of Na^+^ and K^+^ in different ‘Luxiu’/rootstock combinations and organs. Na^+^ content in the roots, stems, and leaves (**A**), K^+^ content in the roots, stems, and leaves (**B**). n=3 for all groups. The bars represent the SE. Bars with different lowercase letters indicate statistically significant differences at *P<0.05* based on one-way ANOVA.

Under salt treatment, L/QAUP-1 showed the highest absorption of Na^+^ and had the strongest ability to transport Na^+^ to the aboveground parts. The Na^+^ of L/QNA201 was mainly concentrated in the roots and was not transported upward. The Na^+^ in L/Qingzhen D1 and L/OHF40 was mainly concentrated in the roots and the lower parts of the grafted part (Figure 7A and Table S2). Only the K^+^ content of L/Qingzhen D1 increased under salt treatment, and it mainly increased in the upper part of grafts; the K^+^ content of the other three grafted seedlings decreased, and the decrease in K^+^ in L/OHF40 was the most significant (*P* < 0.05) (Figure 7B and Table S2).

### Pear clone rootstocks roots and leaves are salt-tolerant by regulateing the transcription of the related genes

**Fig. 8:**
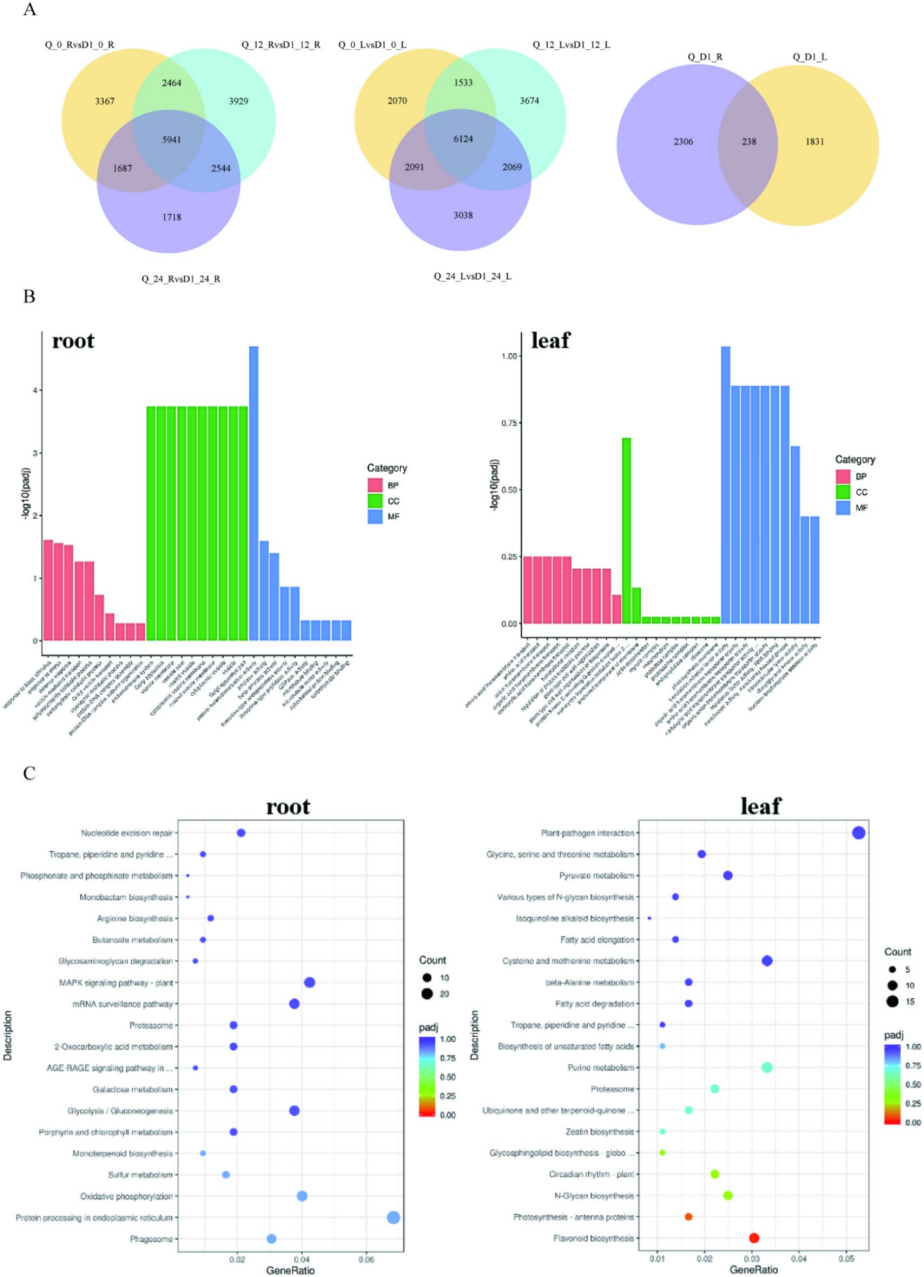
Differentially expressed genes in roots or leaves of QAUP-1 and Anvil D1 under 200 mM salt treatment.Venn diagram of differentially expressed genes (**A**). GO enrichment analysis of differentially expre.sed genes (**B**). KEGG pathway analysis of differentially expressed genes (**C**).

To explore the molecular mechanism of salt tolerance of L/QNA201 and L/Qingzhen D1 we performed RNA sequencing (RNA-seq) analyses to compare the transcriptome changes triggered by treating L/QNA201 and L/Qingzhen D1, in which grafted seedlings was treateded by watering the roots of the rootstocks with 200mM saline. The Venn diagram analysis of differential probe sets is shown as Fig. Among the differential probe sets of L/QNA201 and L/Qingzhen D1 at 200mM.Notably, Qingzhang D1 and QAUP-1 had 2544 differentially expressed genes in roots and 2069 differentially expressed genes in leaves. To further summarized and analyzed the differentially expressed genes of these two parts and it was found that 2306 genes were differentially expressed only in roots, 1831 genes were differentially expressed only in leaves, and 238 genes were differentially expressed in both parts (Figure 8A). To further analyze the distribution of differential gene functions among salt-tolerant rootstocks, GO enrichment analysis was performed on the differential genes. Root differential gene function annotations mainly focused on cellular components, followed by molecular functions. Leaves are mainly focused on molecular functions (Figure 8B).

In order to understand of the biological functions of the differential expression genes, the differential genes were further compared to the KEGG database. the result show that there are 20 pathways with significantly enriched differentially expressed genes (p<0.05). In roots, salt stress mainly affects the processes of protein processing, oxidative phosphorylation and sulfur metabolism in the endoplasmic reticulum. In leaves, it mainly affects flavonoid biosynthesis and photosynthesis(Figure 8C).

The previous study that the salt tolerance of plants is related to the regulation of hormones, transcription factors, ions and antioxidant enzymes. Further analysis found that the expression level of F-box Qingzhen D1, which is a salicylic acid pathway synthesis gene, was higher than that of QAUP-1. In roots, the expression levels of auxin-responsive transcription factor and ethylene-responsive transcription factor ERF were higher than those in QAUP-1, while the expression levels of auxin and ethylene synthesis genes in Qingzhen D1 were lower than those in QAUP-1, The expression levels of transcription factors WRKY and P450 in the leaves and roots of Qingzhen D1 were higher than those of QAUP-1. However, the expression level of MYB was higher in the roots of Qingzhen D1 and higher in the leaves of QAUP-1. The expression of Ca2+ transport-binding pathway genes in QingZhen D1 was higher than that of QAUP-1, but the expression of GNGC (Cyclic nucleotide-gated channels) was the opposite. The expression of APX and POD related to the antioxidant system was higher than that of QAUP-1 in the roots of Qingzhen D1, while the expression of SOD was the opposite (Figure 8C).

## Discussion

This study systematically evaluated the salt tolerance of four pear rootstocks (QNA201, Qingzhen D1, OHF40, QAUP-1) and their regulatory effects on the salt tolerance of grafted ‘Luxiu’ scions. The results showed that there were significant differences in salt adaptability among different rootstocks, and these differences were closely related to their physiological responses and molecular regulatory patterns under salt stress.

The most direct manifestation of salt tolerance among the four rootstocks was the salt injury index. QNA201 and Qingzhen D1 showed only slight leaf damage under both 100 mM and 200 mM NaCl treatments, while QAUP-1 and OHF40 suffered severe damage, confirming that QNA201 and Qingzhen D1 are excellent salt-tolerant rootstocks. Experiments demonstrated that these differences were associated with ion distribution and content. Ion homeostasis regulation is crucial for the accumulation and distribution patterns of Na⁺ under salt stress. QNA201 and Qingzhen D1 retained most of the Na⁺ in their roots, with only a small amount transported to the leaves, which can reduce Na⁺ toxicity to photosynthetic tissues^52^. In addition, QNA201 and Qingzhen D1 could maintain or even increase K⁺ content under high-salt conditions, which might alleviate Na⁺-induced osmotic stress by stabilizing cell membrane potential and enzyme activity. This indicates that salt-tolerant rootstocks can protect the photosynthetic system from salt damage. In this experiment, the SPAD values and photosynthetic parameters of QNA201 and Qingzhen D1 remained relatively stable under salt stress, while those of QAUP-1 and OHF40 decreased more significantly, which is consistent with the findings in mango that salt-tolerant rootstocks maintain photosynthetic function by limiting Na⁺ uptake in leaves^53^.

The salt tolerance of grafted ‘Luxiu’ seedlings depends on rootstock genotypes. L/QNA201 and L/Qingzhen D1 showed the least salt damage, while L/QAUP-1 was the most sensitive. This consistency between the salt tolerance of rootstocks and grafted seedlings confirms that rootstocks play a dominant role in regulating the salt tolerance of scions, and the underlying mechanisms may include three aspects: ion transport coordination—grafted seedlings inherit the Na⁺ transport characteristics of rootstocks, and L/QNA201 and L/Qingzhen D1 retain most of the Na⁺ in roots and the lower part of the graft union, reducing Na⁺ toxicity in leaves and providing photosynthate guarantee for growth under salt stress, which is similar to the mechanism in watermelon and soybean where rootstocks control Na⁺ levels in scions transport^4,54^. In contrast, L/QAUP-1 accumulates a large amount of Na⁺ in leaves, leading to severe scorching. Enhancement of antioxidant systems—L/QNA201 and L/Qingzhen D1 exhibit higher SOD, POD, and CAT activities under salt stress, which can effectively scavenge reactive oxygen species (ROS), consistent with the conclusion that grafted plants adapt to salt stress through antioxidant enzymes^55^.

Transcriptome data revealed the molecular basis of salt tolerance in QNA201 and Qingzhen D1. GO and KEGG analyses showed tissue-specific regulation of their gene expression. Differentially expressed genes in roots are mainly enriched in pathways such as endoplasmic reticulum protein processing and oxidative phosphorylation, indicating that roots support physiological processes such as ion transport and ROS scavenging by enhancing protein folding stability and energy supply. Differentially expressed genes in leaves are concentrated in pathways of flavonoid biosynthesis and photosynthesis; flavonoids, as antioxidants, can synergize with antioxidant enzymes to protect leaves, while the stable expression of photosynthesis-related genes explains the stability of SPAD values and photosynthetic rates in leaves of salt-tolerant rootstocks. In addition, the high expression of F-box genes related to salicylic acid synthesis in Qingzhen D1, as well as the differential expression of transcription factors such as WRKY and P450, is consistent with their known functions in plant stress adaptation^56^.

## Conclusions

Through the observation of morphological parameters and the measurement of physiological and biochemical parameters of 4 pear clonal rootstocks and their grafted seedlings after salt treatment, it was found that the salt tolerance of grafted seedlings was positively correlated with the salt tolerance of rootstocks, and the influence mechanism of 4 rootstocks on the salt tolerance of grafted seedlings was different: L/QingzhenD1 inhibited the upward transport capacity of Na^+^ and enhanced the absorption and upward transport of K^+^. L/QNA201 can inhibit both Na^+^ uptake and upward transport. L/OHF40 has a weak ability to absorb and transport Na^+^, but it can reduce K^+^ content in plants.. L/QAUP-1 has strong Na^+^ absorption and upward transport capacity.

## Supplementary Information

Supplementary Information.

## Data Availability

The raw sequence data reported in this paper have been deposited in the Genome Sequence Archive^57^ in National Genomics Data Center^58^, China National Center for Bioinformation/Beijing Institute of Genomics, Chinese Academy of Sciences (GSA: CRA024546) that are publicly accessible at [https://ngdc.cncb.ac.cn/gsa]. The datasets generated during and analysed during the current study are available from the corresponding author(Ran Wang E-mail:qauwr@126.com) on reasonable request.
