# Supplementary material for: Effect of salt stress tolerance of four pear rootstock clones on the salt resistance of their grafted and the involvement of possible mechanisms
Source: Sci Rep. 2026 Jan 14;16:1890. doi: 10.1038/s41598-025-31476-2 (PMC12804771; doi:10.1038/s41598-025-31476-2)
Supplement: Supplementary file 1 — Supplementary Information. [file 41598_2025_31476_MOESM1_ESM.docx]

**Supplementary appendices**

Table S1 p-value of rootstock

| Analysis of Variance of Morpho-Physiological Characteristics of Four Pear Rootstocks Under Salinity Stress | | |
| --- | --- | --- |
| Source of Variation DF SPAD | df | F |
| 0 mM Salt Treatment Time | 3 | 10.185 * |
| Rootstock variety | 3 | 202.005** |
| 0 mM Salt Treatment Time * Rootstock variety | 9 | 15.463** |
| Error | 48 | 3.247 |
| CV | 3.466 |  |
| Source of Variation DF SPAD |  |  |
| 100 mM Salt Treatment Time | 3 | 71.921** |
| Rootstock variety | 3 | 453.769** |
| 100 mM Salt Treatment Time * Rootstock variety | 9 | 28.277** |
| Error | 48 | 2.826 |
| CV | 3.443 |  |
| Source of Variation DF SPAD |  |  |
| 200 mM Salt Treatment Time | 3 | 30.613 ** |
| Rootstock variety | 3 | 847.886 ** |
| 200 mM Salt Treatment Time * Rootstock variety | 9 | 63.172 ** |
| Error | 48 | 3.331 |
| CV | 3.701 |  |
| Source of Variation DF PN |  |  |
| 0 mM Salt Treatment Time | 3 | 9.678 ** |
| Rootstock variety | 3 | 241.350** |
| 0 mM Salt Treatment Time * Rootstock variety | 9 | 8.454** |
| Error | 32 | 2.020 |
| CV | 8.599 |  |
| Source of Variation DF PN |  |  |
| 100 mM Salt Treatment Time | 3 | 20.160 * |
| Rootstock variety | 3 | 352.184** |
| 100 mM Salt Treatment Time * Rootstock variety | 9 | 28.277** |
| Error | 32 | 6.145 |
| CV | 15.401 |  |
| Source of Variation DF PN |  |  |
| 200 mM Salt Treatment Time | 3 | 73.824** |
| Rootstock variety | 3 | 796.127** |
| 200 mM Salt Treatment Time * Rootstock variety | 9 | 12.018** |
| Error | 32 | 1.909 |
| CV | 9.832 |  |
| Source of Variation DF ET |  |  |
| 0 mM Salt Treatment Time | 3 | 0.086 |
| Rootstock variety | 3 | 20.748** |
| 0 mM Salt Treatment Time * Rootstock variety | 9 | 0.305 |
| Error | 32 | 0.170 |
| CV | 9.276 |  |
| Source of Variation DF ET |  |  |
| 100 mM Salt Treatment Time | 3 | 0.794 |
| Rootstock variety | 3 | 25.438** |
| 100 mM Salt Treatment Time * Rootstock variety | 9 | 1.182* |
| Error | 32 | 0.403 |
| CV | 15.004 |  |
| Source of Variation DF ET |  |  |
| 200 mM Salt Treatment Time | 3 | 22.958** |
| Rootstock variety | 3 | 12.885** |
| 200 mM Salt Treatment Time * Rootstock variety | 9 | 1.426** |
| Error | 32 | 0.112 |
| CV | 11.993 |  |
| Source of Variation DF gs |  |  |
| 0 mM Salt Treatment Time | 3 | 492.201 |
| Rootstock variety | 3 | 66016.799** |
| 0 mM Salt Treatment Time * Rootstock variety | 9 | 456.683 |
| Error | 32 | 695.365 |
| CV | 13.066 |  |
| Source of Variation DF gs |  |  |
| 100 mM Salt Treatment Time | 3 | 8026.524** |
| Rootstock variety | 3 | 55373.344** |
| 100 mM Salt Treatment Time * Rootstock variety | 9 | 3297.088** |
| Error | 32 | 371.398 |
| CV | 11.028 |  |
| Source of Variation DF gs |  |  |
| 200 mM Salt Treatment Time | 3 | 40595.139** |
| Rootstock variety | 3 | 45414.556** |
| 200 mM Salt Treatment Time * Rootstock variety | 9 | 2571.003** |
| Error | 32 | 333.016 |
| CV | 13.277 |  |

|  | NaCl concentration |  | F | P |
| --- | --- | --- | --- | --- |
| Na+ content | 0mM | leaf | 19.429 | 0.0001 |
|  |  | stem | 5.166 | 0.013 |
|  |  | root | 36.731 | 0.0001 |
|  | 100mM | leaf | 53.44 | 0.0001 |
|  |  | stem | 90.938 | 0.0001 |
|  |  | root | 11.086 | 0.0032 |
|  | 200mM | leaf | 242.996 | 0.0001 |
|  |  | stem | 5.766 | 0.0176 |
|  |  | root | 4.679 | 0.036 |
| K+ content | 0mM | leaf | 14.097 | 0.0001 |
|  |  | stem | 47.936 | 0.0001 |
|  |  | root | 16.303 | 0.0001 |
|  | 100mM | leaf | 32.575 | 0.0001 |
|  |  | stem | 80.35 | 0.0001 |
|  |  | root | 2.582 | 0.1261 |
|  | 200mM | leaf | 13.956 | 0.0015 |
|  |  | stem | 23.558 | 0.0003 |
|  |  | root | 14.279 | 0.0014 |

Table S2 p-value of grafted seedlings

| Analysis of Variance of Morpho-Physiological Characteristics of Four Pear Rootstocks Under Salinity Stress | | |
| --- | --- | --- |
| Source of Variation DF SPAD |  |  |
| 0 mM Salt Treatment Time | 3 | 1.890 |
| Grafting Combination | 3 | 7.848 |
| 0 mM Salt Treatment Time * Grafting Combination | 9 | 6.575 |
| Error | 32 | 8.308 |
| CV | 7.851 |  |
| Source of Variation DF SPAD |  |  |
| 200 mM Salt Treatment Time | 3 | 140.280** |
| Grafting Combination | 3 | 234.410** |
| 200 mM Salt Treatment Time * Grafting Combination | 9 | 20.429* |
| Error | 32 | 7.746 |
| CV | 8.622 |  |
| Source of Variation DF SOD |  |  |
| Salt Treatment Time | 3 | 50919.330** |
| Grafting Combination | 3 | 21309.094* |
| Salt Treatment Time * Grafting Combination | 9 | 28609.752** |
| Error | 32 | 5837.579 |
| CV | 29.975 |  |
| Source of Variation DF POD |  |  |
| Salt Treatment Time | 3 | 9128.766** |
| Grafting Combination | 3 | 8217.907** |
| Salt Treatment Time * Grafting Combination | 9 | 3778.821** |
| Error | 32 | 69.515 |
| CV | 13.018 |  |
| Source of Variation DF CAT |  |  |
| Salt Treatment Time | 3 | 258359.655** |
| Grafting Combination | 3 | 209257.106** |
| Salt Treatment Time * Grafting Combination | 9 | 272443.188** |
| Error | 32 | 2782.473 |
| CV | 10.553 |  |

| indexes | NaCl concentration |  | F | P |
| --- | --- | --- | --- | --- |
| Na+ content | 0mM | leaf | 1.371 | 0.3196 |
|  |  | turnk of scion | 26.25 | 0.0002 |
|  |  | turnk of rootstock | 36.687 | 0.0001 |
|  |  | root | 410.196 | 0.0001 |
|  | 200mM | leaf | 2297.212 | 0.0001 |
|  |  | turnk of scion | 1286.088 | 0.0001 |
|  |  | turnk of rootstock | 699.675 | 0.0001 |
|  |  | root | 455.481 | 0.0001 |
| K+ content | 0mM | leaf | 18.452 | 0.0006 |
|  |  | turnk of scion | 96.06 | 0.0001 |
|  |  | turnk of rootstock | 130.661 | 0.0001 |
|  |  | root | 237.31 | 0.0001 |
|  | 200mM | leaf | 408.324 | 0.0001 |
|  |  | turnk of scion | 419.647 | 0.0001 |
|  |  | turnk of rootstock | 800.993 | 0.0001 |
|  |  | root | 121 | 0.0001 |
